# Diastolic Heart Failure Predicted by Left Atrial Expansion Index in Patients with Severe Diastolic Dysfunction

**DOI:** 10.1371/journal.pone.0162599

**Published:** 2016-09-13

**Authors:** Shih-Hung Hsiao, Kuan-Rau Chiou

**Affiliations:** 1 Division of Cardiology, Department of Internal Medicine, E-Da Hospital, I-Shou University, Kaohsiung, Taiwan, Republic of China; 2 School of Medicine, National Yang-Ming University, Taipei, Taiwan, Republic of China; 3 Division of Cardiology, Department of Internal Medicine, Kaohsiung Veterans General Hospital, Kaohsiung, Taiwan, Republic of China; Osaka University Graduate School of Medicine, JAPAN

## Abstract

**Background:**

Left atrial (LA) echocardiographic parameters are increasingly used to predict clinically relevant cardiovascular events. The study aims to evaluate the LA expansion index (LAEI) for predicting diastolic heart failure (HF) in patients with severe left ventricular (LV) diastolic dysfunction.

**Methods:**

This prospective study enrolled 162 patients (65% male) with preserved LV systolic function and severe diastolic dysfunction (132 grade 2 patients, 30 grade 3 patients). All patients had sinus rhythm at enrollment. The LAEI was calculated as (Vol_max_ - Vol_min_) x 100% / Vol_min_, where Vol_max_ was defined as maximal LA volume and Vol_min_ was defined as minimal volume. The endpoint was hospitalization for HF withp reserved LV ejection fraction (HFpEF).

**Results:**

The median follow-up duration was 2.9 years. Fifty-four patients had cardiovascular events, including 41 diastolic and 8 systolic HF hospitalizations. In these 54 patients, 13 in-hospital deaths and 5 sudden out-of-hospital deaths occurred. Multivariate analyses revealed that HFpEF was associated with LAEI.and atrial fibrillation during follow-up. For predicting HFpEF, the LAEI had a hazard ratio of 1.197per 10% decrease. In patients who had HFpEF events, the LAEI significantly (*P*< 0.0001) decreased from 69±18% to 39±11% during hospitalization. Although the LAEI improved during follow-up (53±13%), it did not return to baseline.

**Conclusions:**

The LAEI predicts HFpEF in patients with severe diastolic dysfunction; it worsens during HFpEF events and partially recovers during followup.

## Introduction

Approximately 50% of patients with heart failure (HF) have a normal or near normal left ventricular (LV) ejection fraction (LVEF) [1].Symptoms/signs and physiologic and neurohormonal phenotypes of HF with preserved LVEF (HFpEF) resemble those of HF with reduced LVEF (HFrEF) [2–4].The prognosis of patients with HFpEF is only marginally better than that of patients with HFrEF.Left ventricular diastolic dysfunction with normal systolic function and no HF symptoms, which is classified as stage B (asymptomatic structural heart disease) in the American College of Cardiology/American Heart Association schema,is associated with structural abnormalities and development of HF and is predictive of all-cause mortality [5–7].Diastolic dysfunction, which is usually assessed by Doppler measurements of mitral inflow and tissue Doppler imaging [8, 9], is a well-established predictor of HF.

Left atrial (LA) echocardiographic parameters have an increasingly important role in predicting relevant clinical events [10]. LA dilation is the surrogate of diastolic dysfunction with elevation of LV filling pressure since the left atrium has poor compliance with LV during mitral valve opening in the diastolic phase. The LA diameter has a strong correlation with the severity of LA fibrosis [11], which causes dysfunctional atrial contraction and correlates with the presence and persistence of atrial fibrillation (Af).Because Af complicates and increases the severity of diastolic dysfunction, Af is an indicator of poor prognosis [12]. Compared to other clinical and echocardiographic measurements,the LA expansion index (LAEI) is reportedly superior for predicting LV filling pressure and Af in different subsets [13–17].This study tested the hypothesis that LAEI is a useful predictor of the progression from severe diastolic dysfunction to HFpEF.

## Materials and Methods

### Study population

The study cohort was prospectively enrolled from Mar 21, 2011 to Mar 20, 2013. Patients were recruited from the special outpatient HF clinic at Kaohsiung Veterans General Hospital–a tertiary center in Taiwan.Patients were invited to participate in the study if they had preserved LVEF that met the criteria for severe diastolic dysfunction and sinus rhythm. Exclusion criteria were lack of informed consent or any history of the following: 1) HF hospitalization, 2) prosthetic mitral valve or mitral stenosis, mitral regurgitation or aortic regurgitation of moderate or higher severity, 3) any atrial septal abnormality (*e*.*g*., defect or aneurysm), 4) rhythm other than sinus rhythm, and 5) inadequate image quality.The study protocol was approved by institutional review board of Kaohsiung Veterans General Hospital. Patients invited to participate in this study were enrolled only after giving written informed consent.

### Echocardiography

Echocardiography (iE33 system; Philips Medical System, Andover, Massachusetts) was performed in left decubitus position to maximize image quality. Trans-mitral flow profiles, including peak early-diastolic flow velocity (E), late-diastolic flow velocity (A), and mitral early deceleration time (DT), were assessed. The LVEF was calculated using Simpson biplane technique. In Doppler echocardiography, pulmonary arterial systolic pressure was estimated by using the modified Bernoulli equation to calculate the right ventricular to right atrial pressure gradient during systole (i.e., 4V^2^, where V is the velocity of the tricuspid regurgitation jet in m/s). Right atrial pressure was then estimated according to echocardiographic characteristics of the inferior vena cava, assigned a standardized value [18], and added to the calculated gradient. The LV mass was calculated using the formula described by Devereux and Reichek [19]. The LV mass was indexed to body surface area (BSA). Pulsed-wave tissue Doppler imaging (TDI) was performed using spectral pulsed Doppler signal filters with the Nyquist limit set to 15–20 cm/s and with the optimal gain set to minimum. In apical views, a pulsed-wave Doppler sample volume was placed at the level of the mitral annulus over the septal and lateral borders. Pulsed-wave TDI results were characterized by a myocardial systolic wave (s’) and 2 diastolic waves: early (e’) and atrial contraction (a’). A pulsed-wave TDI tracing recorded over 5 cardiac cycles at a sweep speed of 100 mm/s was used for offline calculations. The E/e’ method was used to estimated LV filling pressure based on the average e’ of septal and lateral mitral annuli [20]. Preserved LV systolic function was defined as LVEF more than 50%. Diastolic dysfunction was assessed as described previously [8, 9, 21]. The presence of mitral E/A < 0.75 or DT > 240 ms was considered evidence of impaired relaxation. In more severe stages of diastolic dysfunction with pseudonormal LV filling, trans-mitral flow characteristics resemble those in patients with normal diastolic function. This study defined both pseudonormal and normal LV filling as mitral E/A of 0.75 to 1.50 and DT of 151 to 240 ms, differentiated by E/e’ (> 10 for pseudonormal). Restrictive diastolic filling, which was the most severe diastolic dysfunction, was associated with markedly elevated LV filling pressure (LVFP). The presence of mitral E/A > 1.5 or DT ≦ 150 ms was considered evidence of this abnormality. Severe diastolic function was classified as pseudonormal/restrictive filling of mitral inflow and E/e’ more than 15, indicating LVFP more than 15 mmHg. Histories of hyperlipidemia, hypertension, and smoking were recorded by the examining physicians. Diabetes mellitus was defined according to American Diabetes Association criteria [22]. Creatinine clearance (CCr) was estimated by Cockroft-Gault equation according to baseline weight and serum creatinine. Renal dysfunction was defined as CCr < 60 ml/min at enrollment [23].

### LA volume parameter measurements

All volume measurements were calculated by biplane area-length method in apical four- and two-chamber views [24]. The LA volumes were measured at two points: immediately before mitral valve opening (maximal LA volume or Vol_max_) and at mitral valve closure (minimal LA volume or Vol_min_). The LAEI was calculated as (Vol_max_—Vol_min_) x 100% / Vol_min_ and LA emptying fraction as (Vol_max_—Vol_min_) x 100% / Vol_max_. In all patients, LA volumes were indexed to BSA [17]. The ratio of Vol_max_ to septal a’ was also measured because it indicates severe diastolic dysfunction [25].

### Clinical and echocardiographic follow-up

Participants were followed up at our HF clinic every 3 months for at least 2 years. The primary endpoint was hospitalization with HFpEF. The secondary endpoint was any cardiovascular event, including hospitalization for HFEF, hospitalization for HFpEF, or death related to cardiovascular disease. This study focused on hospitalization with HFpEF. HF hospitalization was defined as a hospital admission due to symptoms fitting New York Heart Association (NYHA), class III or IV, signs of elevated jugular venous pressure, pulmonary rales, and third heart sound, and chest radiography showing pulmonary edema. These clinical signs and symptoms had been accompanied by either failing cardiac output or pulmonary edema treated with intravenous diuretics, inotropes, or vasodilators. The adjudication considered any available supportive documentation of an increased pulmonary capillary wedge pressure, decreasing oxygen saturation, and end organ hypoperfusion. Based on LVEF during hospitalization, HF was further classified as HFrEF or HFpEF. An LVEF less than 45% at event was classified as HFrEF. For a tachyarrhythmia-related transient decrease in LVEF, HF was still considered an HFpEF event if LV systolic function fully recovered after control of heart rate or rhythm was established. Patients with HF events received additional echocardiography checkups during admissions and 3 months after events. Patients without events received echocardiography checkups annually. In patients lost to follow up, research assistants contacted and interviewed patients by telephone or, if necessary, by visiting patients at their homes. Death was certified by death records, death certificates, or hospital medical records.

### Identification of predisposing factors

All patients hospitalized for HF received 24-hour ECG monitoring for 3–5 days and routine ECG checkups at admission and discharge. The ECG findings were used to determine the type and severity of arrhythmia. Conditions such as infection, gastrointestinal bleeding, myocardial infarction, etc. were classified as predisposing factors.

### Interobserver variability

In the first 50 enrolled cases, LA parameters (including Vol_max_, Vol_min_, LA emptying fraction, and LAEI) were measured by two independent observers. Interobserver variability was calculated as the difference between the values obtained by the two observers divided by the mean. Interobserver difference and variability were 2.7 ± 3.5 ml/m^2^ and 4.7 ± 7.3% for Vol_max_ and 1.6 ± 2.2 ml/m^2^ and 5.7 ± 7.2% for Vol_min_, respectively. Therefore, interobserver variability in LA emptying fraction and LAEI measurements were 5.6 ± 7.2% and 4.7 ± 7.4%, respectively.

### Statistical analysis

The SPSS software was used for all statistical analyses. All continuous variables were presented as means ± standard deviation. A *P* value of < 0.05 was considered statistically significant. Clinical characteristics were compared by chi-square analysis of categorical variables. Cox proportional hazards regression was used to examine associations among clinical conditions, echocardiographic parameters and cardiovascular events. The independent prognostic value was determined by incremental multivariate models adjusted for covariates showing significant (*P* < 0.05) associations with events in univariate analysis. The area under receiver-operating characteristic (ROC) curve (AUROC) was used to evaluate the sensitivity and specificity of predictors of HFpEF events. Based on multivariate Cox regression after adjusting potential confounders, 3-year cumulative event-free survival was estimated according to LAEI.

## Results

### Basic characteristics

In total, 194 patients with severe diastolic dysfunction were enrolled. Thirty-two were excluded due to refusal to participate or due to history of severe mitral regurgitation, HF hospitalization or Af. Thus, 162 patients were analyzed. Table 1 shows the causes of severe diastolic dysfunction (132 grade 2 patients and 30 grade 3 patients). During a mean follow-up duration of 2.9 years, 54 patients (33.3%) had cardiovascular events, including 49 HF hospital admissions (41 HFpEF, 8 HFrEF). During follow-up, 13 patients died during hospitalization (10 with HFpEF, 3 with HFrEF), and five died suddenly while living at home or in a nursing home. Table 2 compares the basic characteristics according to HFpEF after excluding other cardiovascular events not associated with HFpEF (S1 Table for all cardiovascular events). Compared to the non-HFpEF group, the event group had more patients with female gender, hypertension, and renal dysfunction. During follow-up, the event group also had a higher severity of LV hypertrophy, a higher severity of LA dilation, a lower LAEI, and a higher frequency of Af.

**Table 1:** Causes of severe diastolic dysfunction.

Cause of severe diastolic dysfunction	Number
Coronary artery disease	44
Hypertensive cardiovascular disease	43
Aortic stenosis	25
Uremic cardiomyopathy	22
Hypertrophic cardiomyopathy	18
Apical hypertrophic cardiomyopathy	4
Restrictive cardiomyopathy (all amyloidosis)	2
Unknown	4

**Table 2:** Comparisons according to diastolic heart failure.

Variable	No event (N = 108)	Diastolic HF (N = 41)	*P* values
Age (year)	64±17	69±15	0.102
Gender (male/female)	70/38	21/20	0.041
Diabetes (%)	13 (12%)	7 (17.1%)	0.219
Hypertension (%)	42 (28.9%)	27 (65.9%)	<0.0001
Current tobacco use (%)	29 (26.9%)	12 (29.3%)	0.418
Baseline NYHA functional class			0.594
I	5/108 (4.6%)	1/41 (2.4%)	
II	103/108(95.4%)	40/41(97.6%)	
III	0/108 (0%)	0/41 (0%)	
IV	0/108 (0%)	0/41 (0%)	
Hemoglobin (gm/dl)	11.2±3.6	10.9±4.1	0.468
Baseline B-type Natriuretic peptide (pg/ml)	108±86	121±109	0.362
Coronary artery disease (%)	25 (23.1%)	13 (31.7%)	0.125
Renal dysfunction (%)	24 (22.2%)	22 (53.9%)	<0.0001
Dyslipidemia (%)	68 (63%)	28 (68%)	0.276
Systolic blood pressure (mmHg)	138±21	141±20	0.089
Heart rate (BPM)	70±14	75±15	0.043
Medications at baseline			
Aspirin	32 (30%)	16 (39%)	0.046
Beta-blocker	26 (24%)	21 (51%)	<0.0001
Calcium channel blocker	13 (12%)	12 (29%)	<0.0001
Angiotensin-converting enzyme inhibitor/receptor blocker	12 (11%)	4 (10%)	0.497
Diuretics	25 (23%)	17 (41%)	0.009
Statins	51 (47%)	20 (49%)	0.542
Interventricular septum (mm)	12.6±2.1	13.5±2.3	0.02
Diastolic left ventricular internal diameter (mm)	46±4	47±5	0.721
Systolic left ventricular internal diameter (mm)	26±5	27±5	0.67
Early-diastolic mitral inflow (cm/s)	105±23	125±27	<0.0001
Late-diastolic mitral inflow (cm/s)	71±21	85±27	0.001
Deceleration time (ms)	155±37	154±35	0.721
Left ventricular ejection fraction (%)	60±4	58±5	0.195
Pulmonary artery systolic pressure (mmHg)	41±11	45±14	0.027
LV mass index (g/m^2^)	151±39	177±53	0.002
RV—s' (cm/s)	11.8±3.3	11.5±3.7	0.559
RV—e' (cm/s)	9.8±4.0	8.6±3.3	0.075
RV—a' (cm/s)	11.9±3.7	11.8±4.0	0.847
Septal—s' (cm/s)	7.3±1.9	6.7±1.8	0.104
Septal—e' (cm/s)	6.9±2.7	5.6±2.0	0.008
Septal—a' (cm/s)	8.0±2.5	6.9±2.9	0.017
Lateral—s' (cm/s)	8.1±2.2	8.0±2.3	0.743
Lateral—e' (cm/s)	9.0±3.3	7.1±2.4	0.002
Lateral—a' (cm/s)	8.5±2.5	7.9±3.2	0.237
E/e'	15.8±6.4	17.8±7.1	0.007
Maximal indexed LA volume (ml/m^2^)	43±22	55±24	0.004
Minimal indexed LA volume (ml/m^2^)	21±13	30±14	0.033
LA expansion index (%)	111±57	69±18	<0.0001
LA emptying fraction (%)	48.6±21.2	45.8±18.1	0.193
Maximal indexed LA volume/a'	6.3±4.4	9.8±4.9	0.001
Atrial fibrillation during follow-up (%)	16 (14.8%)	25 (61%)	<0.0001
Event–HFpEF	0	41	
Event–HFrEF	0	0	
Event–death	0	10	

**a’**: late-diastolic velocity of annulus; **E/e’:** early-diastolic mitral inflow divided by the average of septal and lateral mitral annular velocities; **e’**: early-diastolic velocity of annulus; **HFpEF**: heart failure with preserved left ventricular systolic function; **HFrEF**: heart failure with reduced left ventricular systolic function; **LA**: left atrium; **Lateral**: lateral mitral annulus; **LV**: left ventricle; **MPI**: myocardial performance index derived by tissue Doppler; **NYHA**: New York Heart Association; **RV**: right ventricle; **s’**: systolic velocity of annulus; Septal: septal mitral annulus;

(Analyses are performed after excluding these events which are not due to diastolic heart failure).

### Predisposing factors for HFpEF events

The leading predisposing factor for adverse events was atrial tachy-arrhythmia (Table 3). Of 41 events, 28 were associated with tachy-arrhythmia (25 with Af, 1 with atrial tachycardia, 1 with atrial flutter, and 1 with paroxysmal supraventricular tachycardia) Five events had unknown causes. Only 3 of 25 patients with Af regained sinus rhythm at discharge.

**Table 3:** Predisposing factors of diastolic heart failure.

Predisposing factors of diastolic heart failure*	Number
Atrial tachy-arrhythmia	28
Myocardial ischemia	8
Sepsis/ infection	3
Gastrointestinal tract bleeding	2
Hypertension emergency/crisis	2
Aortic dissection	1
Unknown	5

*Atrial tachy-arrhythmia: 1 case with atrial tachycardia, 1 case with atrial flutter, 1 case with paroxysmal supraventricular tachycardia, 25 cases with atrial fibrillation;

Myocardial ischemia: 2 cases withatrial fibrillation; Sepsis/infection: 2 cases with atrial fibrillation; 1 case with atrial flutter; Gastrointestinal tract bleeding: 2 cases with atrial fibrillation; Hhypertension crisis: 1 case with atrial fibrillation.

### Univariate and multivariate analyses of HFpEF predictors

**Fig 1:**
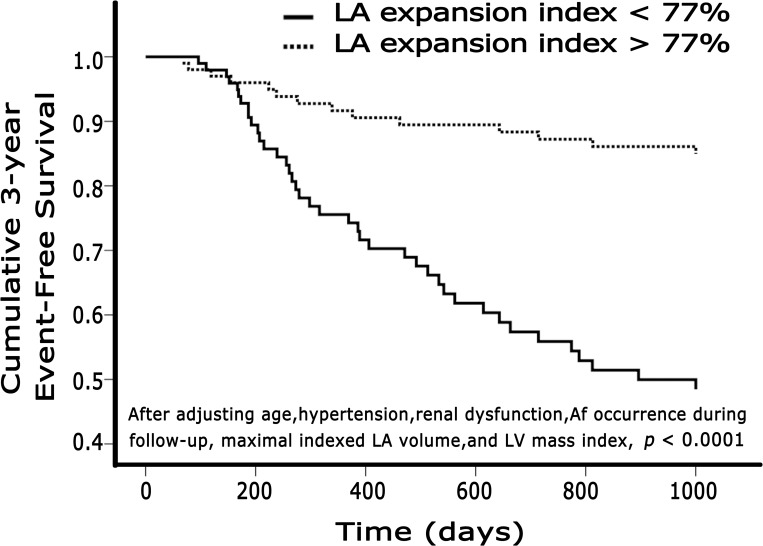
Cox proportional regression curves according to LA expansion index. The cumulative 3-year event-free survival rate by the Cox proportional hazards regression results according to LAEI after adjusting for age, hypertension, renal dysfunction, LV mass index, Vol_max_ and Af occurrence during follow-up.

Table 4 shows the results of univariate and multivariate analyses of HFpEF predictors (S2 Table for all cardiovascular events). Multivariate analysis showed that the only independent prognosticators were LAEI and Af during follow-up. For predicting HFpEF, LAEI had a hazard ratio of 1.197 per 10% decrease. The ROC curve analysis showed that an LAEI less than 77% was the best cut-off point for predicting HFpEF with AUROC 0.786, sensitivity 74%, and specificity 72%. Fig 1 shows the Cox proportional hazards regression results according to LAEI after adjusting for age, hypertension, renal dysfunction, LV mass index, Vol_max_ and Af occurrence during follow-up.

**Table 4:** Univariate and multivariate analyses for the predictors of diastolic heart failure.

	Univariate analysis		Multivariate analysis	
Variables	Hazard ratio	*P* values	Hazard ratio	*P* values
	(95% CI)		(95% CI)	
Age (years)	1.016 (0.996–1.036) per 1 year increase	0.121		
Female gender	1.576 (0.854–2.909)	0.145		
Diabetes	1.170 (0.519–2.639)	0.706		
Hypertension	2.583 (1.354–4.927)	0.004	1.840 (0.899–3.765)	0.095
Renal dysfunction	3.112 (1.683–5.754)	<0.0001	1.935 (0.954–3.926)	0.068
Atrial fibrillation during follow-up period	5.875 (3.131–11.024)	<0.0001	3.505 (1.671–7.355)	0.001
Left ventricular ejection fraction (%)	0.969 (0.912–1.029) per 1% increase	0.3		
Maximal indexed LAV (ml/m^2^)	1.016 (1.005–1.028) per 1 ml/m^2^ increase	0.005	0.995 (0.973–1.017) per 1 ml/m^2^ increase	0.631
Minimal indexed LAV (ml/m^2^)	1.017 (1.000–1.034) per 1 ml/m^2^ increase	0.052		
LA expansion index (%)	1.255 (1.129–1.396) per 10% decrease	<0.0001	1.197 (1.045–1.371) per 10% decrease	0.009
E/e'	1.058 (1.020–1.098) per 1 unit increase	0.003	1.012 (0.983–1.099) per 1 unit increase	0.114
Maximal indexed LAV/ a'	1.087 (1.038–1.138) per 1 unit increase	<0.0001	0.998 (0.914–1.089) per 1 unit increase	0.958
LV mass index (g/m^2^)	3.112 (1.683–5.754) per 1 g/m^2^ increase	<0.0001	1.006 (0.998–1.014) per 1 g/m^2^ increase	0.153

Abbreviations as shown in **Table 2.**

### Changes in LAEI by longitudinal follow-up

**Fig 2:**
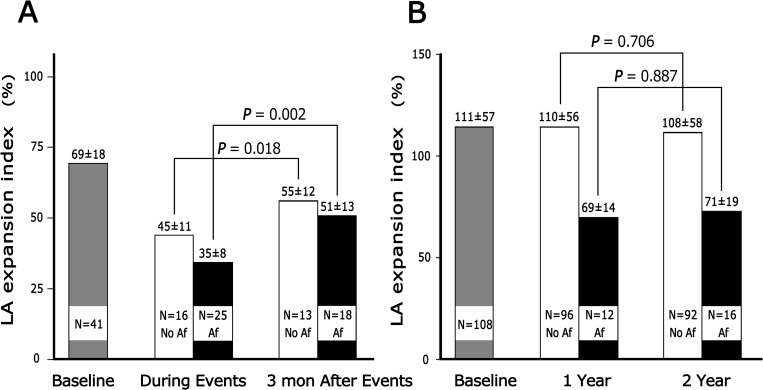
The temporal changes in LAEI by longitudinal follow-up. (A) Temporal changes in left atrial expansion index at baseline (69 ± 18%), during diastolic heart failure (HFpEF) admission (all cases 39 ± 11%; HF without atrial fibrillation (Af) 45 ± 11%, HF with Af 35 ± 8%) and 3 months after HFpEF admission (all cases 53 ± 13%; No Af 55 ± 12%, Af 5 ± 13%) in patients with adverse events; (B) Annual follow up of LA expansion index in patients without HFpEF.

Fig 2 shows the temporal changes in LAEI by longitudinal follow-up. In the HFpEF group, LAEI during hospitalization decreased from 69 ± 18% (N = 41) to 45 ± 11% in patients without Af (N = 16) and to 35 ± 8% in patients with Af (N = 25). The LAEI significantly improved 3 months later (from 45 ± 11% to 55 ± 12% in patients without Af, *P =* 0.018; from 35 ± 8% to 51 ± 13% in patients with Af, *P =* 0.002) but did not return to baseline level. Of 108 patients without HFpEF, 16 developed Af during the follow-up period. During follow up, the LAEI did not substantially change in patients without Af (111 ± 57% at enrollment; 110 ± 56% at year 1; 108 ± 58% at year 2 follow up) or in those with subsequent occurrence of Af (69 ± 14% at year 1; 71 ± 19% at year 2 follow-up).

### Univariate and multivariate analyses of predictors of subsequent Af

Af was the most common predisposing factor for HFpEF. S3 Table shows the results of univariate and multivariate analyses. Only renal dysfunction, Vol_max_, and LAEI were independent predictors of subsequent Af. For predicting the further occurrence of Af, the LAEI had a hazard ratio of 1.209 per 10% decrease.

## Discussion

Whereas our previous study showed that LAEI is useful for predicting HFrEF and all-cause mortality [26], this study further showed that LAEI is associated with HFpEF events in patients with severe diastolic dysfunction and that LAEI corresponds with dynamic changes in diastolic function and disease course. It also indicates that, once diastolic dysfunction progresses to HFpEF, LAEI inevitably declines until diastolic dysfunction is irreversible. Therefore, identifying diastolic dysfunction and preventing its progression to HFpEF is crucial for improving prognosis in these patients. In our prior study [15], LAEI more than 150% is almost associated with normal subjects. The majority of patients with LAEI less than 50% have severe diastolic dysfunction with either pseudonormal or restrictive patterns of mitral inflow. The gray zone of LAEI between 50% and 150% indicates that patients overlaying with mild to moderate diastolic dysfunction. In the study cohorts, LAEI is better than LA size for predicting HF events (Table 4) in line with our previous study [26], but the relationship of LA size and LAEI merits further investigation.

### Prognostic indicators of events

Af was the leading predisposing factor for HFpEF events in this study. In line with our prior study [27], LAEI was an independent predictor of further occurrence of Af (S3 Table). Atrial fibrosis is an underlying cause of the occurrence and persistence of Af [28]. Af can be viewed by 2 different aspects. First, it represents the severity of atrial fibrosis and underlying LV filling dysfunction, which is directly related to diastolic function. Secondly, Af with rapid ventricular response shortens diastolic phase worsening LV diastolic filling, which exacerbates diastolic dysfunction even more. In the general population, renal dysfunction is a significant prognostic indicator of further events [29]. According to a sub-study of the VALIANT trial, renal dysfunction is also a major cardiovascular risk factor and is associated with higher than normal baseline LA volume, LV mass, and LV mass/LV end-diastolic volume ratio [30]. This suggests that renal dysfunction is closely related to myocardial geographic changes, which are associated with a high probability of further events. In line with the report of Kane et al. [31], E/e’ didn’t play a significant role in the progression from diastolic dysfunction to HFpEF. Age, diabetes, and coronary artery disease did not reach statistical significance (Tables 2 and 4). A possible explanation is selection bias since only patients with severe diastolic dysfunction were enrolled; those with mild diastolic dysfunction were excluded.

### LAEI associated with HFpEF event

Although severe diastolic dysfunction itself indicates a high HF risk, studies of HFpEF predictors in these patients are scarce [31, 32]. In this study, 25.3% (41/162) patients suffered HFpEF, of which 24.4% (10/41) died during hospitalization. Table 4 shows that, compared to Doppler measurements of mitral inflow (E/A wave) and TDI (E/e'), LA volume is a better predictor of event rate. One explanation is that the enrollment criteria for the study cohort included E/A wave and E/e’. The pathophysiology of severe diastolic dysfunction involves a complex interaction of multiple systemic factors. The LAEI reflects Af severity and LV filling pressure and predicts probability of subsequent Af. The LAEI is also useful for identifying patients in whom diastolic dysfunction is likely to progress to HFpEF. A community-based survey performed by Aljaroudi et al. revealed that patients with severe diastolic dysfunction had an 18% chance of recovering normal diastolic function [7]. In our study cohort, only 8 of 162 (4.9%) patients showed an improvement in diastolic function from pseudonormal to impaired relaxation during follow up. All had improved LAEI and were event-free, which suggests that severe diastolic dysfunction may be reversible.

### Course of LAEI during HFpEF event

Fig 2 shows that LAEI deteriorated during HFpEF hospitalization and that recovery was incomplete during follow-up. During HFpEF, a large reduction in LAEI is expected because poor compliance of the LV causes the LA to stiffen. After HFpEF is resolved, outpatients showed significantly improved LAEI. However, the LAEI was still lower than that at baseline, which suggests that, once diastolic dysfunction progresses to HFpEF, a downhill trend is eventually inevitable. Further studies in larger populations are needed to determine whether aggressive managements of underlying diseases can modify the natural course of the disease, whether such managements are still effective in patients who already have end-stage diastolic dysfunction, and how soon interventions for underlying diseases should be performed to prevent progression of diastolic dysfunction.

### Special concerns of Af and heart rate

Most occurrences of HFpEF in this study were induced by tachyarrhythmia (68.3%), particularly Af. Fig 2 shows that 16 patients in the non-event group had Af but not HFpEF. Their ventricular rates were significantly lower than those in HFpEF patients with Af (85 ± 37 vs. 138 ± 42, respectively; *P* < 0.0001). The ventricular rate of Af could be the major cause of the different results. However, one unanswered question is whether the difference in LAEI between the two groups (69 ± 14% vs. 35 ± 8%, respectively; *P* < 0.0001) could be induced by tachycardia only. In HFpEF cases with Af, LAEI significantly improved from 35 ± 8% to 51 ± 13%, and the ventricular rate reached 87 ± 28 beats per minute in recovery phase. Interestingly, the significant difference in LAEI between the two groups (69 ± 14% vs. 51 ± 13%, respectively; *P* = 0.011) indicated that an underlying diastolic dysfunction and disease course, not Af or tachycardia, was the cause of distinct LAEI. Further large-scale studies are needed to clarify the interaction between heart rate and LAEI and the relationship between Af and LAEI.

### Study limitations

This study has several limitations. First, this study analyzed data collected at a single tertiary center, which may have resulted in selection bias since the number of patients was relatively small. Second, the underlying causes of severe diastolic dysfunction were quiet diverse and the progression of underlying diseases and diastolic dysfunction were fairly different. However, this study indicates that the application of LAEI is suitable to diverse diseases with severe diastolic dysfunction in real-world practice. Third, this study did not address whether serial decreases in LAEI increase power to predict further progression of diastolic dysfunction. Additionally, although LAEI can identify patients with a high probability of diastolic dysfunction progressing to HFpEF, and although some studies suggest aggressive treatment of underlying diseases in early phase to eliminate further HFpEF [7], the best method of preventing HFpEF is debatable, and many predisposing factors are apparently unpreventable. Third, since other echocardiographic measures of LA function such as segmental atrial function, strain, strain rate, and atrial response to exercise were not examined, so we couldn’t compare the efficiency and predicting power of LAEI to those of strain rate/strain.

## Conclusion

LAEI predicts HFpEF in patients with severe diastolic dysfunction, and worsens during HFpEF events and recovers partially during follow-up.

## Supporting Information

S1 Table Comparisons according to all cardiovascular events. (DOC)

S2 Table Univariate and multivariate analyses for all cardiovascular events. (DOC)

S3 Table Univariate and multivariate analyses for the predictors of the presence of atrial fibrillation. (DOC)
