# Supplementary material for: Diastolic Heart Failure Predicted by Left Atrial Expansion Index in Patients with Severe Diastolic Dysfunction
Source: PLoS One. 2016 Sep 13;11(9):e0162599. doi: 10.1371/journal.pone.0162599 (PMC5021281; doi:10.1371/journal.pone.0162599)
Supplement: S1 Table — (DOC) [file pone.0162599.s001.doc]

**S1**Table. Comparisons according to all cardiovascular events

| **Variable** | **No event (N=108)** | **Event**  **(N=54)** | **p values** |
| --- | --- | --- | --- |
| Age (year) | 64±17 | 68±15 | 0.082 |
| Gender (male/female) | 70/38 | 28/26 | 0.036 |
| Diabetes (%) | 13 (12%) | 12 (22.2%) | 0.035 |
| Hypertension (%) | 42 (28.9%) | 33 (61.1%) | 0.001 |
| Current tobacco use (%) | 29 (26.9%) | 16 (29.6%) | 0.381 |
| Coronary artery disease (%) | 25 (23.1%) | 19 (35.2%) | 0.061 |
| S/P bypass surgery | 4/25 (16%) | 3/19 (15.8%) | 0.813 |
| Renal dysfunction (%) | 24 (22.2%) | 26 (48.1%) | <0.0001 |
| Dyslipidemia (%) | 68 (63%) | 34 (63%) | 0.892 |
| Systolic blood pressure (mmHg) | 138±21 | 143±24 | 0.021 |
| Heart rate (BPM) | 70±14 | 76±14 | 0.009 |
| Interventricular septum (mm) | 12.6±2.1 | 13.3±2.2 | 0.065 |
| Diastolic left ventricular internal diameter (mm) | 46±4 | 47±5 | 0.213 |
| Systolic left ventricular internal diameter (mm) | 26±5 | 28±5 | 0.058 |
| Early-diastolic mitral inflow (cm/s) | 105±23 | 122±28 | <0.0001 |
| Late-diastolic mitral inflow (cm/s) | 71±21 | 82±27 | 0.002 |
| Deceleration time (ms) | 155±37 | 153±31 | 0.615 |
| Left ventricular ejection fraction (%) | 60±4 | 58±6 | 0.124 |
| Pulmonary artery systolic pressure (mmHg) | 41±11 | 43±13 | 0.019 |
| LV mass index (g/m^2^) | 151±39 | 174±48 | 0.002 |
| Medications at baseline |  |  |  |
| Aspirin | 32 (30%) | 24 (44%) | 0.005 |
| Beta-blocker | 26 (24%) | 28 (52%) | <0.0001 |
| Calcium channel blocker | 13 (12%) | 17 (31%) | <0.0001 |
| Angiotensin-converting enzyme inhibitor/receptor blocker | 12 (11%) | 7 (13%) | 0.276 |
| Diuretics | 25 (23%) | 24 (44%) | 0.003 |
| Statins | 51 (47%) | 26 (48%) | 0.877 |
| RV - s' (cm/s) | 11.8±3.3 | 11.3±3.7 | 0.358 |
| RV - e' (cm/s) | 9.8±4.0 | 8.6±3.1 | 0.073 |
| RV - a' (cm/s) | 11.9±3.7 | 11.8±4.0 | 0.834 |
| Septal - s' (cm/s) | 7.3±1.9 | 6.7±1.9 | 0.068 |
| Septal - e' (cm/s) | 6.9±2.7 | 5.8±2.5 | 0.016 |
| Septal - a' (cm/s) | 8.0±2.5 | 6.8±2.8 | 0.008 |
| Lateral - s' (cm/s) | 8.1±2.2 | 7.9±2.6 | 0.64 |
| Lateral - e' (cm/s) | 9.0±3.3 | 7.3±2.7 | 0.001 |
| Lateral - a' (cm/s) | 8.5±2.5 | 8.1±3.1 | 0.317 |
| E/e' | 15.8±6.4 | 17.6±7.4 | 0.125 |
| Maximal indexed LA volume (ml/m^2^) | 43±22 | 52±22 | 0.014 |
| Minimal indexed LA volume (ml/m^2^) | 22±13 | 29±13 | 0.03 |
| LA expansion index (%) | 111±57 | 66±20 | <0.0001 |
| LA emptying fraction (%) | 48.6±21.2 | 45.1±16.9 | 0.086 |
| Maximal indexed LA volume/a' | 6.3±4.4 | 9.2±5.0 | 0.001 |
| Atrial fibrillation during follow-up (%) | 16 (14.8%) | 27 (50%) | <0.0001 |
| Event -- diastolic heart failure | 0 | 41 (75.9%) |  |
| Event -- systolic heart failure | 0 | 8 (14.8%) |  |
| Event -- death | 0 | 18 (33.3%) |  |

Abbreviations as shown in **Table 2**
