# Supplementary material for: Diastolic Heart Failure Predicted by Left Atrial Expansion Index in Patients with Severe Diastolic Dysfunction
Source: PLoS One. 2016 Sep 13;11(9):e0162599. doi: 10.1371/journal.pone.0162599 (PMC5021281; doi:10.1371/journal.pone.0162599)
Supplement: S2 Table — (DOC) [file pone.0162599.s002.doc]

**S2** Table. Univariate and multivariate analyses for all cardiovascular events

|  | **Univariate analysis** |  | **Multivariate analysis** |  |
| --- | --- | --- | --- | --- |
|  |  |  |  |  |
| **Variables** | **Hazard ratio** | ***P* values** | **Hazard ratio** | ***P* values** |
|  | **(95% CI)** |  | **(95% CI)** |  |
| Age (years) | 1.015 (0.998-1.033) per 1 year increase | 0.083 |  |  |
| Female gender | 1.592 (0.934-2.717) | 0.088 |  |  |
| Diabetes | 1.798 (0.946-3.417) | 0.073 |  |  |
| Hypertension | 2.052 (1.187-3.547) | 0.01 | 1.484 (0.803-2.741) | 0.207 |
| Renal dysfunction | 2.569 (1.505-4.385) | 0.001 | 1.904 (1.015-3.570) | 0.045 |
| Atrial fibrillation during follow-up period | 3.689 (2.166-6.313) | <0.0001 | 2.063 (1.082-3.934) | 0.028 |
| Left ventricular ejection fraction (%) | 0.952 (0.899-1.007) per 1% increase | 0.084 |  |  |
| Maximal indexed LAV (ml/m^2^) | 1.011 (1.001-1.022) per 1 ml/m^2^ increase | 0.03 | 0.984 (0.969-1.022) per 1 ml/m^2^ increase | 0.426 |
| Minimal indexed LAV (ml/m^2^) | 1.015 (1.000-1.029) per 1 ml/m^2^ increase | 0.052 |  |  |
| LA expansion index (%) | 1.317 (1.144-1.528) per 10% decrease | <0.0001 | 1.216 (1.137-1.435) per 10 % decrease | <0.0001 |
| E/e' | 1.028 (0.991-1.006) | 0.136 |  |  |
| Maximal indexed LAV/ a' | 1.087 (0.964-1.138) per 1 unit increase | 0.089 |  |  |
| LV mass index (g/m^2^) | 1.010 (1.004-1.015) per 1 g/m^2^ increase | 0.001 | 1.009 (1.002-1.016) per 1 g/m^2^ increase | 0.007 |

Abbreviations as shown in **Table 2**
