# Supplementary material for: Diastolic Heart Failure Predicted by Left Atrial Expansion Index in Patients with Severe Diastolic Dysfunction
Source: PLoS One. 2016 Sep 13;11(9):e0162599. doi: 10.1371/journal.pone.0162599 (PMC5021281; doi:10.1371/journal.pone.0162599)
Supplement: S3 Table — (DOC) [file pone.0162599.s003.doc]

**S3 Table. Univariate and multivariate analyses for the predictors of the presence of atrial fibrillation**

|  | **Univariate analysis** |  | **Multivariate analysis** |  |
| --- | --- | --- | --- | --- |
|  |  |  |  |  |
| **Variables** | **Hazard ratio** | ***P* values** | **Hazard ratio** | ***P* values** |
|  | **(95% CI)** |  | **(95% CI)** |  |
| Age (years) | 1.021 (1.001-1.042) per 1 year increase | 0.043 | 1.011 (0.989-1.034) per 1 year increase | 0.319 |
| Female gender | 1.272 (0.697-2.322) | 0.434 |  |  |
| Diabetes | 1.285 (0.596-2.771) | 0.522 |  |  |
| Hypertension | 0.817 (0.449-1.485) | 0.507 |  |  |
| Renal dysfunction | 2.166 (1.189-3.945) | 0.012 | 1.902 (1.009-3.584) | 0.047 |
| Left ventricular ejection fraction (%) | 1.015 (0.959-10.74) per 1% increase | 0.606 |  |  |
| Maximal indexed LA volume (ml/m^2^) | 1.025 (1.014-1.035) per 1 ml/m^2^ increase | <0.0001 | 1.027 (1.001-1.054) per 1 ml/m^2^ increase | 0.043 |
| Minimal indexed LA volume (ml/m^2^) | 1.041 (1.024-1.058) per 1 ml/m^2^ increase | <0.0001 | 0.988 (0.945-1.032) per 1 ml/m^2^ increase | 0.576 |
| LA expansion index (%) | 1.226 (1.177-1.324) per 10% decrease | <0.0001 | 1.209 (1.153-1.311) per 10% decrease | 0.034 |
| E/e' | 0.997 (0.954-1.042) per 1 unit increase | 0.906 |  |  |
| Maximal indexed LA volume/a' | 1.042 (0.934-1.257) per 1 unit increase | 0.121 |  |  |
| LV mass index (g/m^2^) | 1.006 (1.000-1.012) per 1 g/m^2^ increase | 0.052 | 1.006 (0.998-1.014) per 1 g/m^2^ increase | 0.153 |

Abbreviations as shown in **Table 2**
